# Preoperative Anxiety and Depression Are Associated with Lateral Compartment Osteoarthritis Progression After Medial Unicompartmental Knee Arthroplasty: A Retrospective Observational Cohort Study

**DOI:** 10.3390/jcm15176568

**Published:** 2026-08-26

**Authors:** Zheng Wang, Mincong Du, Zheng Li, Shuai An, Guanglei Cao, Ye Huang

**Affiliations:** 1 Department of Knee Preservation Surgery, Beijing Jishuitan Hospital, Capital Medical University, 68 Huanan North Road, Huilongguan Street, Changping District, Beijing 102200, China; wangzheng@jst-hosp.com.cn (Z.W.); dmc57@163.com (M.D.); 2 Department of Orthopedics, Xuanwu Hospital, Capital Medical University, 45 Changchun Street, Xicheng District, Beijing 100053, China; xwgkl@163.com (Z.L.); anshuai102@hotmail.com (S.A.)

**Keywords:** anxiety/depression, lateral compartment osteoarthritis progression, medial unicompartmental knee arthroplasty

## Abstract

**Background:** This study aimed to evaluate whether preoperative anxiety and/or depression (AD) is associated with mid-term lateral compartment osteoarthritis progression (LOP) after medial unicompartmental knee arthroplasty (mUKA). **Methods:** This retrospective observational cohort study analyzed a prospectively maintained institutional database of consecutive patients who underwent mUKA between March 2016 and December 2020. Preoperative psychological status was assessed using the Hospital Anxiety and Depression Scale (HADS); patients with subscore ≥8 were categorized as having AD. Clinical outcomes included Oxford Knee Score (OKS), Forgotten Joint Score (FJS), Kujala score, and complications at a mean follow-up of 70 months. Serial full-length standing radiographs were used to measure coronal alignment and Kellgren–Lawrence (K/L) grade in the lateral compartment. LOP was defined as an increase of ≥1 K/L grade between the early postoperative and final follow-up radiographs. Univariate and multivariable logistic regression were used to identify factors associated with LOP. **Results:** A total of 203 knees met the inclusion criteria; 26 knees were classified into the AD group and 177 into the Non-AD group. At final follow-up, the AD group had worse OKS, smaller OKS improvement, and lower FJS and Kujala scores. Radiographic follow-up was available in 99 knees. LOP occurred in 26.3% of knees overall. The rate of LOP was significantly higher in the AD group than in the Non-AD group (60.0% vs. 20.2%). In univariate analysis, AD was associated with LOP. In multivariable logistic regression, preoperative AD remained associated with a higher likelihood of LOP after adjustment for prespecified covariates. **Conclusions:** Preoperative anxiety and/or depression was associated with a higher frequency of radiographic lateral compartment osteoarthritis progression and worse mid-term patient-reported outcomes after mUKA. These findings should be considered exploratory and hypothesis-generating.

## 1. Introduction

Psychological factors have emerged as important determinants of outcome in orthopedic surgery over the past decade [1]. Specific psychological traits such as pain catastrophizing, maladaptive coping, and low self-efficacy have been associated with poorer perceived surgical benefit and less satisfactory recovery [2]. Consistent with these observations, psychological distress has been linked not only to inferior functional recovery and reduced health-related quality of life following total knee arthroplasty (TKA) [3,4,5], but also to a higher risk of postoperative complications such as periprosthetic joint infection, wound complications, and aseptic loosening [6]. However, it is uncertain whether these observations can be directly generalized to unicompartmental knee arthroplasty (UKA). UKA differs from TKA in surgical invasiveness and soft-tissue preservation [7], implant mechanics, and typical patient demographics and indications [8]. Thus, the effect of psychological distress on postoperative recovery and complication profiles observed in TKA may not apply to UKA.

Medial UKA (mUKA) provides reliable pain relief, functional improvement, and durable survivorship in appropriately selected patients with end-stage medial compartment knee osteoarthritis (KOA) [9,10,11]. Nevertheless, mUKA is not exempt from failure. After aseptic loosening, lateral compartment osteoarthritis progression (LOP) represents the second most common mode of failure, accounting for roughly 19% of all revisions and up to 25–34% in early series [12,13,14]. Proposed contributors to LOP include coronal overcorrection of varus deformity toward valgus alignment, imbalance in coronal soft-tissue tension (including overstuffing of the medial compartment), and persistent loading patterns that increase contact stress in the lateral compartment [15,16,17,18].

Beyond mechanical factors, psychological distress may also contribute to postoperative radiographic progression through interacting behavioral and biological pathways [19]. Psychological distress has been associated with fear-avoidance behavior, altered gait patterns, and increased muscle co-contraction, all of which may modify dynamic tibiofemoral loading during daily activities [20]. In addition, depression has been linked to chronic low-grade systemic inflammation, whereas inflammatory activation has been implicated in KOA progression [21,22]. Although these mechanisms have not been directly investigated following mUKA, they provide a biologically plausible rationale for evaluating whether preoperative anxiety and/or depression (AD) is associated with subsequent LOP.

Previous studies have primarily focused on the association between preoperative AD and postoperative pain, satisfaction, and patient-reported outcomes after knee arthroplasty [23]. However, whether psychological distress is also associated with radiographic progression of the non-resurfaced lateral compartment after mUKA remains unknown. To our knowledge, no previous study has specifically investigated this association. The objective of the present study was to determine whether preoperative AD is associated with postoperative LOP after mUKA in a cohort of patients with a mean follow-up of 70 months.

## 2. Materials and Methods

### 2.1. Study Design and Cohort

This retrospective observational cohort study was conducted using a prospectively maintained institutional database of consecutive patients who underwent mUKA at our institution between March 2016 and December 2020. The study protocol was approved by the institutional ethics committee, and all procedures complied with the Declaration of Helsinki. A total of 226 patients (252 knees) met the initial screening criteria. All patients were of East Asian ancestry and Han Chinese ethnicity. All procedures were performed by a single senior orthopedic surgeon experienced in UKA, using the same implant system (Oxford Phase III mobile-bearing prosthesis, twin-peg femoral component; MP instrumentation; Biomet UK Ltd., Waterton Industrial Estate, Bridgend, UK). This study is reported in accordance with the Strengthening the Reporting of Observational Studies in Epidemiology (STROBE) Statement. A completed STROBE checklist is provided as Supplementary Material.

**Figure 1:**
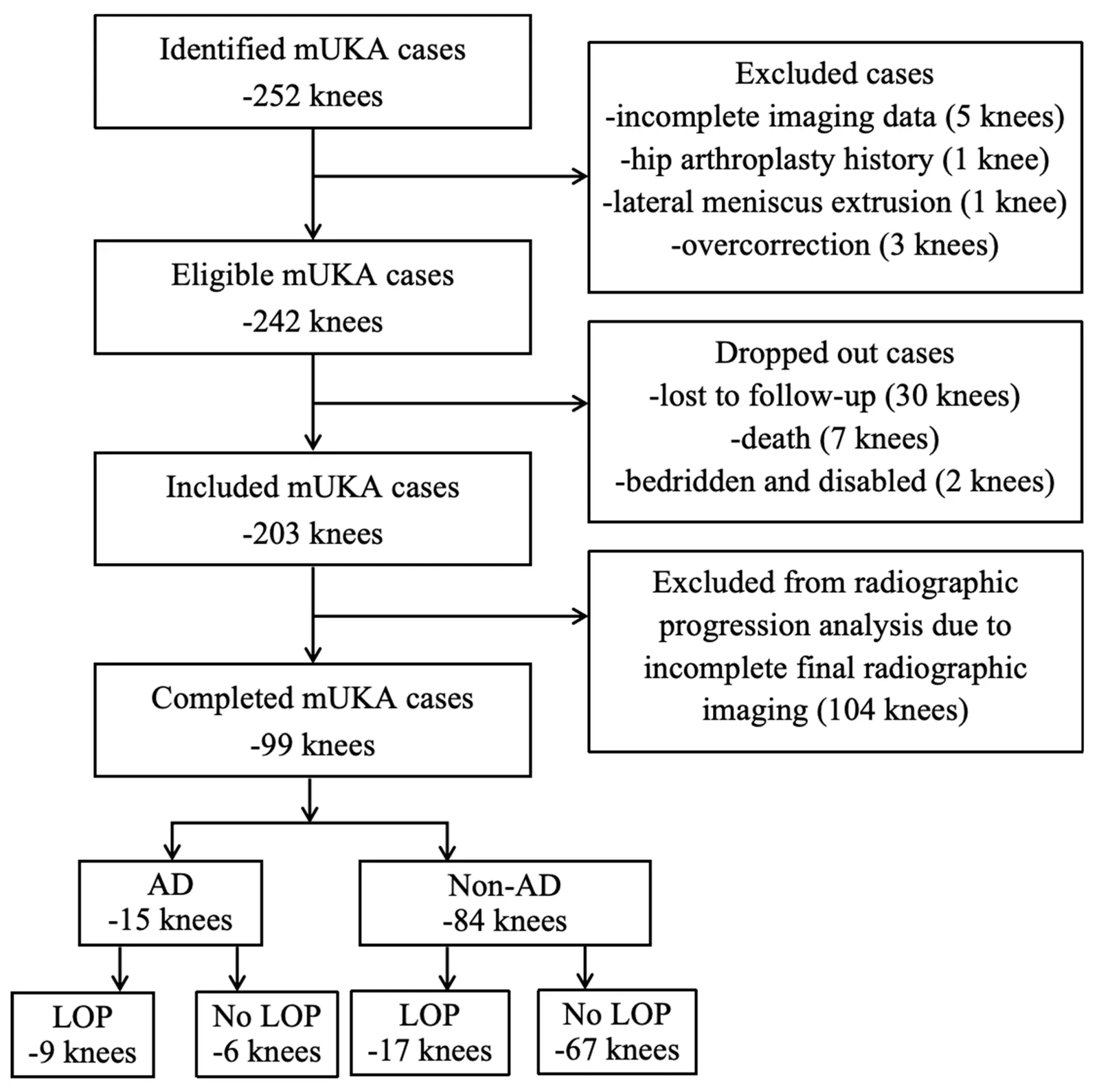
Flowchart illustrating patient selection, application of eligibility criteria, follow-up, and the final cohorts included in the clinical and radiographic analyses. mUKA, medial unicompartmental knee arthroplasty; AD, anxiety and/or depression; LOP, lateral compartment osteoarthritis progression.

The eligibility criteria used before cohort inclusion are summarized in Table 1. Two exclusion criteria were applied specifically to minimize confounding in the assessment of lateral compartment degeneration: (i) preoperative compromise of the lateral meniscus, and (ii) excessive coronal overcorrection after UKA. The rationale for these exclusions was that both pre-existing lateral meniscal dysfunction and excessive valgus correction could accelerate degeneration of the lateral compartment and therefore obscure the association of interest in this study. In addition, overcorrection was defined according to published methodology as postoperative mechanical hip–knee–ankle angle (mHKA) exceeding the calculated arithmetic hip–knee–ankle angle (aHKA) by >3° [24]. Subsequent losses during follow-up are presented separately in the study flowchart (Figure 1). Baseline demographic and clinical characteristics, including age, sex, body mass index, diagnosis, surgical side, coronal alignment parameters, and follow-up duration, were collected from the prospectively maintained institutional database.

### 2.2. Assessment of Anxiety and Depression

Psychological status was evaluated using the Hospital Anxiety and Depression Scale (HADS) [25]. HADS consists of two subscales: anxiety (HADS-A) and depression (HADS-D), each comprising seven items scored from 0 to 3, yielding a maximum subscale score of 21. Scores of 8–11 are typically interpreted as borderline abnormal, and scores > 11 as indicative of clinically relevant anxiety or depression [26]. In accordance with the prior literature [27], we classified a HADS-A or HADS-D score ≥ 8 as the presence of anxiety or depression, respectively. The validated Chinese version of HADS was routinely administered within one week before surgery as part of the standard preoperative assessment. Patients with elevated scores on either subscale were categorized as the anxiety and/or depression (AD) group, and patients with scores < 8 on both subscales were categorized as the non-anxiety/depression (Non-AD) group. None of the patients received treatment for AD within our institution during the study period. No patients had documented severe psychiatric disorders that precluded routine preoperative assessment or surgery.

### 2.3. Radiological and Clinical Outcome Measures

**Figure 2:**
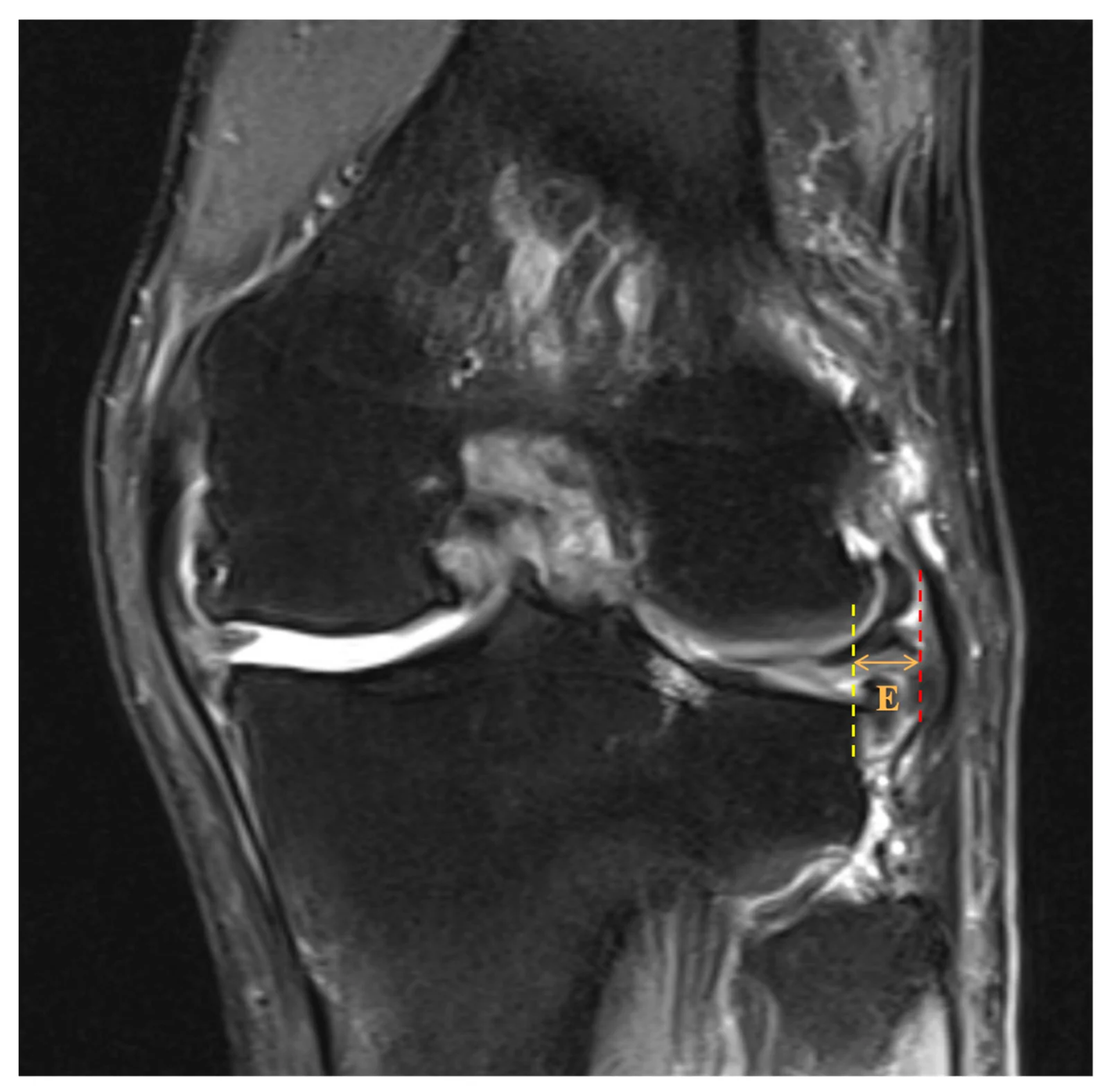
Representative coronal proton density fat-suppressed MRI demonstrating lateral meniscus extrusion (LME). LME was identified as displacement of the peripheral margin of the lateral meniscus beyond the lateral tibial plateau margin. Extrusion distance (E) was measured as the perpendicular distance between the lateral tibial plateau margin (yellow dashed line) and the peripheral margin of the lateral meniscus (red dashed line), representing the extent of lateral meniscus extrusion.

All patients underwent preoperative magnetic resonance imaging (MRI) of the knee (3.0-T system, General Electric, Chicago, IL, USA). Lateral meniscal extrusion was assessed on coronal MRI at the most laterally convex portion of the tibial plateau (Figure 2). On the day of surgery, after induction of anesthesia and prior to arthroplasty implantation, valgus stress radiographs with a radiographic scale were obtained to evaluate the status and reducibility of the lateral compartment [28].

Full-length standing lower-extremity radiographs were acquired within the first postoperative week to document initial coronal alignment. Patients were subsequently encouraged to return for scheduled outpatient follow-up to obtain mid-term radiographic assessments. On these radiographs, the HKA angle was measured, and osteoarthritic change in the lateral compartment was graded according to the Kellgren–Lawrence (K/L) classification. Following the definition proposed by Misir et al. [29], LOP was defined as an increase of at least one K/L grade in the lateral compartment between the early postoperative radiograph and the final follow-up radiograph. Two experienced musculoskeletal radiologists independently evaluated all radiographs while blinded to the clinical information and study groups. Any discrepancies in HKA measurements were averaged after joint review, whereas disagreements in K/L grading were resolved by consensus. If consensus could not be reached, a senior musculoskeletal radiologist made the final determination.

Patient-reported outcomes were collected preoperatively and at final follow-up. The Oxford Knee Score (OKS) was recorded preoperatively and at final follow-up. The Forgotten Joint Score (FJS) and Kujala score were obtained at final follow-up. All perioperative and post-operative complications from the index surgery through final follow-up were documented, including but not limited to wound-related complications, periprosthetic joint infection, urinary tract infection, deep vein thrombosis, periprosthetic fracture, instability or dislocation, and implant loosening. The primary outcome was radiographic LOP. Secondary outcomes included postoperative patient-reported outcome measures (OKS, FJS, and Kujala score) and postoperative complications. Patients underwent routine postoperative follow-up with clinical assessment and radiographic evaluation according to institutional practice.

### 2.4. Statistical Analysis

The normality of continuous variables was assessed using the Shapiro–Wilk test before statistical comparisons. Normally distributed variables are presented as mean ± standard deviation and were compared using Student’s *t*-test, whereas non-normally distributed variables are presented as median (interquartile range) and were compared using the Mann–Whitney U test. Categorical variables are reported as counts with percentages and were compared using the chi-square test or Fisher’s exact test. Analyses involving radiographic LOP were restricted to knees with both early postoperative and final follow-up radiographs. No imputation was performed for missing final radiographic outcomes because K/L-grade progression could not be reliably inferred from the available clinical variables. To assess the potential for selection bias, baseline and clinical characteristics were compared between knees with and without complete serial radiographic follow-up. Univariate analyses were first performed to identify variables associated with LOP. Candidate variables were considered on the basis of clinical relevance, the previous literature, and univariable findings. Given the limited number of LOP events, a restricted set of clinically relevant covariates was included in the primary multivariable logistic regression model to evaluate the adjusted association between AD status and LOP. All statistical analyses were performed using SPSS Statistics (version 25; IBM Corp., Armonk, NY, USA). All *p* values were two-tailed, and statistical significance was defined as *p* < 0.05.

## 3. Results

### 3.1. Cohort Characteristics and Baseline Comparisons

After application of the predefined inclusion and exclusion criteria (Table 1), 203 knees from 177 patients were available for analysis. The cohort included 41 men and 136 women, with a mean age of 68.3 ± 7.1 years, a mean body mass index (BMI) of 27.5 ± 3.6 kg/m^2^, and a mean follow-up duration of 70.8 ± 14.7 months.

Based on preoperative HADS scores, 26 knees were classified into the AD group and 177 knees were classified into the Non-AD group. Patients in the AD group were older than those in the Non-AD group (71.12 ± 7.37 vs. 67.90 ± 6.96 years; *p* = 0.030). BMI, sex distribution, and coronal alignment parameters (preoperative and immediate postoperative HKA angle) did not differ significantly between groups. Mean follow-up duration was comparable (74.27 ± 14.17 vs. 70.25 ± 14.72 months; *p* = 0.194) (Table 2).

### 3.2. Patient-Reported Outcomes and Complications

Preoperative OKS did not differ significantly between the AD and Non-AD groups (26.12 ± 5.88 vs. 24.99 ± 6.08; *p* = 0.377). At approximately 5-year follow-up, patients with AD demonstrated significantly worse subjective outcomes across all instruments. Compared with the Non-AD group, the AD group had a lower postoperative OKS (33.81 ± 6.31 vs. 42.31 ± 3.67; *p* < 0.001), a smaller OKS improvement from baseline (7.69 ± 6.92 vs. 17.32 ± 6.40; *p* < 0.001), a lower FJS (60.69 ± 27.60 vs. 87.94 ± 15.12; *p* < 0.001), and a lower Kujala score (49.69 ± 14.17 vs. 65.85 ± 11.20; *p* < 0.001).

The overall complication rate did not differ significantly between groups (19.2% in the AD group vs. 9.6% in the Non-AD group; *p* = 0.171). The distribution of specific adverse events—including wound-related events, periprosthetic joint infection, urinary tract infection, deep vein thrombosis, periprosthetic fracture, instability, and implant loosening—was similar (Table 3).

### 3.3. Lateral Compartment Radiographic Progression

Complete early postoperative and final follow-up radiographs were available for 99 knees from 99 unique patients, representing 48.8% of the full clinical cohort. To characterize potential differences between knees with and without complete radiographic follow-up, measured baseline characteristics and clinical outcomes were compared (Supplementary Table S1). Although most measured variables were similar between groups, the complete-radiographic-follow-up group had a significantly longer follow-up duration. These comparisons describe measured characteristics only and do not exclude selection bias due to unmeasured factors. Within this radiographic subset (Table 4), the early postoperative K/L grade of the lateral compartment did not differ between groups (*p* = 0.687). By final follow-up, however, the AD group demonstrated a right shift in K/L distribution, including one knee progressing to K/L grade III, whereas most knees in the Non-AD group remained at K/L grades 0–I (*p* = 0.005). Using the predefined criterion of ≥1-grade increase in lateral K/L score, LOP occurred more frequently in the AD group than in the Non-AD group (60.0% [9/15] vs. 20.2% [17/84]; *p* = 0.004). Despite the observed radiographic progression, no knee underwent revision surgery for symptomatic lateral compartment osteoarthritis during the study follow-up.

### 3.4. Characteristics Associated with Radiographic LOP

In univariate comparisons (Table 5), radiographic LOP was associated with the presence of AD (34.6% vs. 8.2%; *p* = 0.004), right-sided surgery (76.9% vs. 46.6%; *p* = 0.008), and a slightly larger immediate postoperative HKA angle (177.13 ± 2.54° vs. 175.67 ± 3.09°; *p* = 0.033). Knees with radiographic LOP also demonstrated a larger HKA angle at final follow-up and poorer patient-reported outcomes, including lower postoperative OKS, smaller OKS improvement, and lower Kujala scores (all *p* ≤ 0.05).

Only clinically relevant baseline and perioperative variables were entered into the multivariable model. In the multivariable model adjusted for clinically relevant covariates (age, surgical side, and postoperative HKA), preoperative AD remained significantly associated with LOP (aOR = 7.453, 95% CI: 2.033–27.082, *p* = 0.003). Right-sided surgery and greater postoperative HKA were also associated with LOP (Right-sided: aOR = 4.180, 95% CI: 1.370–12.751, *p* = 0.012; postoperative HKA: aOR = 1.240, 95% CI: 1.025–1.516, *p* = 0.031), whereas age was not. Sensitivity analyses using expanded, parsimonious, and follow-up-adjusted models yielded consistent findings, with preoperative AD remaining associated with LOP across all model specifications (Supplementary Tables S2 and S3).

## 4. Discussion

The principal finding of this study is that preoperative psychological distress, operationalized as AD, may be associated with both inferior mid-term patient-reported outcomes and a higher likelihood of radiographic LOP following mUKA. Patients with AD reported lower OKS, FJS, and Kujala scores at approximately 70 months, and the rate of radiographic LOP in the AD group was approximately threefold that of the Non-AD group. Importantly, the association between AD and LOP persisted after adjustment for age, surgical side, and postoperative HKA. However, given the limited number of progression events and the wide confidence interval, the magnitude of this association should be interpreted cautiously. These findings suggest that preoperative psychological status may have potential relevance for risk stratification and follow-up after mUKA.

Anxiety and depression are common among older adults and have been associated with functional decline, higher mortality, and increased healthcare utilization [30]. Prior studies in TKA have shown that psychological distress is associated with persistent pain, dissatisfaction, prolonged hospitalization, and higher rates of medical and surgical complications [31,32]. More recently, Ten et al. [23] reported that patients with AD demonstrate worse subjective outcomes both before and after UKA. In our cohort, the prevalence of AD as defined by HADS (12.8%) was comparable to, though slightly lower than, previously reported rates of approximately 14–16% in TKA candidates measured by HADS [33,34]. Consistent with these data, we found that patients with AD experienced less improvement in OKS and reported significantly worse FJS and Kujala scores at mid-term follow-up. The between-group differences at final follow-up were numerically greater than previously reported clinically meaningful thresholds for these instruments, suggesting that the observed differences may also be clinically meaningful. Nevertheless, because these thresholds were primarily established for within-patient longitudinal improvement, they should not be directly extrapolated to cross-sectional between-group comparisons. This is clinically relevant because psychological distress can heighten pain sensitivity, shape pain-related expectations, and impair communication quality and therapeutic alliance between patient and surgeon, all of which may translate to slower functional recovery and lower perceived success [35,36]. Although perioperative complications were numerically more frequent in the AD group, the study may have been underpowered to detect moderate differences. Therefore, the absence of statistical significance should not be interpreted as equivalent complication risk. These observations support further investigation of potentially modifiable psychological factors in the preoperative setting.

The most novel finding of this study is that AD may be associated with radiographic LOP after mUKA. LOP is a well-recognized mode of radiographic progression in mobile-bearing mUKA and remains a leading indication for revision when severe [37]. In our radiographic subset, 26.3% of knees demonstrated a one K/L grade increase in the lateral compartment at follow-up, whereas 73.7% remained stable. This rate of progression is lower than the 34.9% reported by Misir et al. [29]. We believe this difference is partly attributable to our strict exclusion criteria, which were designed to minimize pre-existing risk of lateral compartment overload. Specifically, we excluded cases with postoperative coronal overcorrection, because valgus overcorrection—often due to excessive insert thickness or medial soft-tissue imbalance—has been implicated in increasing lateral compartment contact stress and contributing to structural progression [38]. In our series, postoperative HKA angles in both the LOP and Non-LOP groups remained within mild varus rather than frank valgus, thereby limiting excessive lateral loading. We also excluded patients with preoperative lateral meniscal extrusion on MRI, which is known to compromise load distribution, reduce lateral compartment stability, and act as an independent risk factor for structural progression [39]. By restricting these mechanical confounders, we were able to more directly assess the contribution of psychological distress.

**Figure 3:**
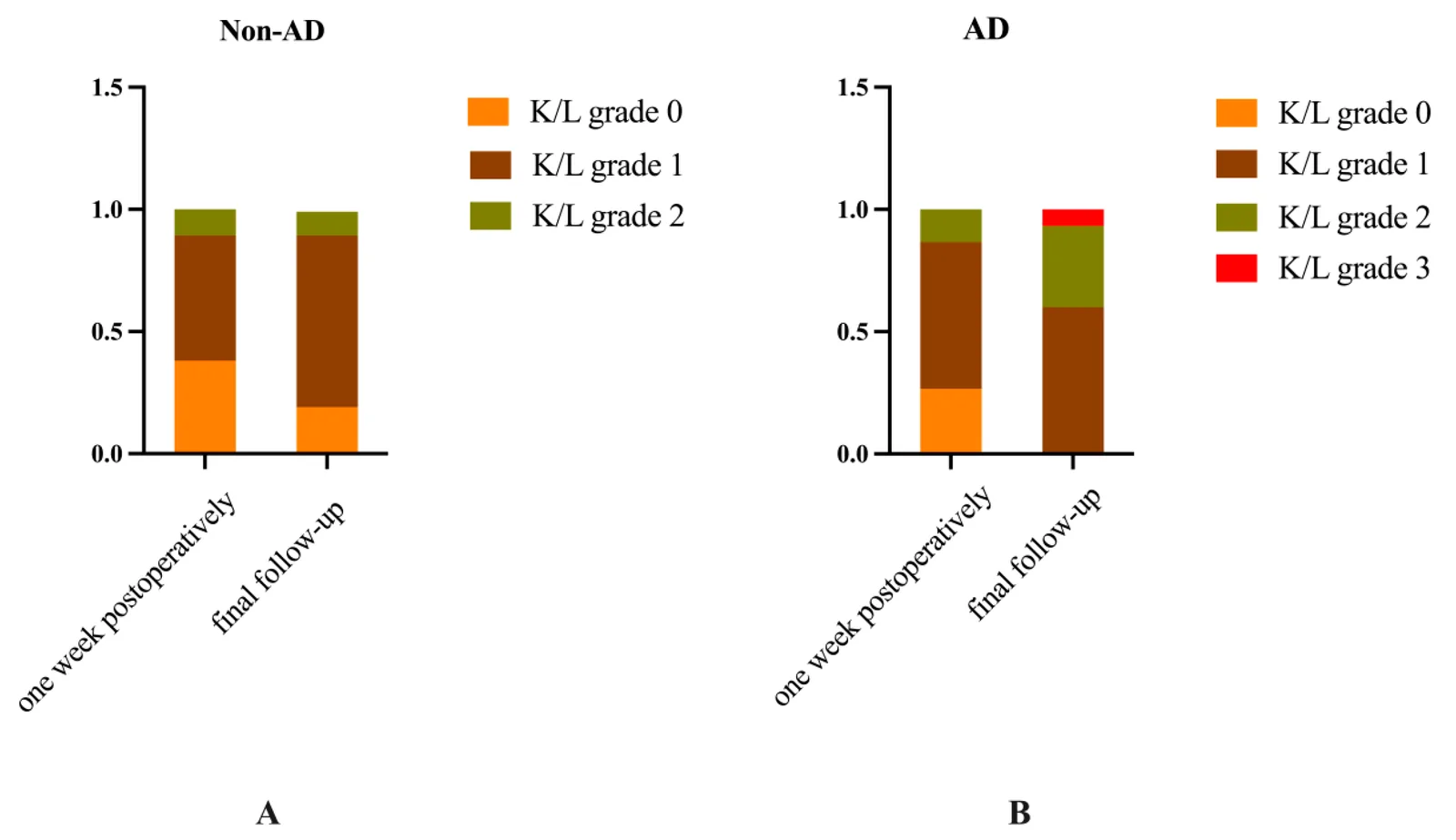
Distribution of lateral compartment Kellgren–Lawrence (K/L) grades at one week postoperatively and at final follow-up according to anxiety and/or depression (AD) status. Panel (**A**) shows the Non-AD group and Panel (**B**) the AD group. Each stacked bar represents the proportion of knees within each K/L grade category, demonstrating changes in radiographic severity over time. AD, anxiety and/or depression; K/L, Kellgren–Lawrence.

Even with comparable early postoperative K/L grades, the AD group demonstrated a rightward shift in K/L distribution by final follow-up (including one case progressing to grade III), whereas most knees in the Non-AD group remained at grades 0/I (Figure 3). Consequently, the observed rate of LOP was significantly higher in the AD group (60.0%) than in the Non-AD group (20.2%). In univariate analyses, LOP was associated with greater immediate postoperative and final HKA angles, consistent with previous evidence linking coronal alignment with LOP [29]. In the multivariable model, preoperative AD remained associated with LOP after adjustment for age, surgical side, and postoperative HKA. Greater postoperative HKA and right-sided surgery were also associated with LOP, whereas age was not. Similar findings were observed in the parsimonious sensitivity analysis. These findings suggest that, even when excessive valgus overcorrection is avoided, both mechanical alignment and patient-level factors may contribute to lateral compartment radiographic progression. LOP after mUKA may therefore reflect an interaction between postoperative biomechanics and patient-specific behavioral or loading characteristics rather than a purely geometric process.

The observed association between AD and LOP may be interpreted through three complementary behavioral and biological pathways. However, these pathways were not directly evaluated in the present study and should therefore be regarded as hypotheses rather than demonstrated mechanisms. First, biobehavioral gait: AD and fear-avoidance behaviors are associated with greater co-contraction of periarticular musculature during gait [40]. Instrumented implant and musculoskeletal modeling studies have demonstrated that increased co-contraction elevates tibiofemoral contact forces, which may shift dynamic load toward the lateral compartment after mUKA [41]. Second, inflammation–neuroendocrine susceptibility: depression is consistently linked to low-grade systemic inflammation, including elevated IL-6 and C-reactive protein [42], while synovitis is known to predict both symptoms and structural progression in KOA [43]. This milieu may reduce the lateral compartment’s tolerance to load, thereby potentially contributing to structural progression under otherwise acceptable alignment. Third, central pain processing: preoperative central sensitization has been associated with worse pain and poorer patient-reported outcomes after arthroplasty and may promote stiff, protective, “guarding” movement patterns that perpetuate abnormal loading [44]. Taken together, these mechanisms provide a biologically plausible basis for the observed association between psychological distress, perceived recovery, and structural progression of the non-resurfaced compartment.

Right-sided surgery was also associated with a higher likelihood of LOP in the revised multivariable model. One plausible interpretation is differential functional demand: the operated limb may represent the patient’s dominant, “working” leg during activities such as stair negotiation, sit-to-stand transitions, and repetitive weight-bearing, thereby experiencing greater cumulative mechanical loading in the lateral compartment. This interpretation is consistent with a previous five- to 10-year follow-up study, which reported that surgery on the dominant limb was independently associated with LOP after mUKA [29]. However, because limb dominance was not systematically recorded in our cohort, it remains uncertain whether the observed association with right-sided surgery truly reflects dominant-limb loading or simply laterality. Therefore, right-sided surgery should be regarded as a surrogate rather than a direct measure of limb dominance. Nevertheless, these findings suggest that LOP after mUKA may be influenced not only by coronal alignment but also by patient-specific loading environments. This interpretation remains exploratory and requires confirmation in future prospective studies incorporating direct assessment of limb dominance.

These findings should be interpreted as hypothesis-generating and identify priorities for future research rather than justify changes in routine clinical practice. Future prospective multicenter studies should determine whether preoperative psychological screening using validated instruments such as HADS provides incremental prognostic value beyond established clinical, radiographic, mechanical, and surgical risk factors. Such studies should concurrently assess psychological distress, pain intensity, central sensitization, gait characteristics, rehabilitation adherence, inflammatory biomarkers, limb dominance, and postoperative alignment to clarify their independent and interactive associations with clinical and radiographic outcomes. Future prospective trials should also evaluate whether perioperative pathways incorporating psychological support, expectation management, graded rehabilitation, and targeted cognitive–behavioral strategies improve patient-reported or radiographic outcomes in patients with preoperative psychological distress. Until such evidence is available, the present findings should not be interpreted as supporting routine psychological screening or modification of standard clinical management after mUKA.

This study has several limitations. First, it is a single-center retrospective analysis, which may introduce selection and information bias; moreover, standardized pain intensity measures, such as VAS or NRS scores, were unavailable in this retrospective cohort. Second, radiographic follow-up was available in only 48.8% of knees, and the primary outcome was based on a one-grade increase in the Kellgren–Lawrence classification, which may be influenced by reader variability, radiographic positioning, image quality, and differences in follow-up duration. The longer follow-up duration in this subgroup may have increased the opportunity to detect radiographic progression. Although an additional sensitivity analysis adjusting for follow-up duration yielded similar findings, residual selection bias cannot be excluded. Third, combining AD into a single exposure group may have obscured potential differences between these psychological conditions. Information regarding psychological or pharmacological treatment received outside our institution was also unavailable and may therefore represent residual confounding. Furthermore, although the primary radiographic analysis included one knee per patient, bilateral knees in the secondary clinical analyses may have introduced modest within-patient correlation. Finally, only 26 LOP events were available for multivariable analysis. Although the number of covariates was deliberately restricted and sensitivity analyses yielded consistent results, the model remains susceptible to overfitting and residual confounding. Therefore, the adjusted associations should be interpreted as exploratory and require confirmation in larger prospective cohorts.

## 5. Conclusions

Preoperative anxiety and/or depression was associated with worse mid-term patient-reported outcomes and a higher rate of radiographic lateral compartment osteoarthritis progression after mUKA. However, given the retrospective design, incomplete radiographic follow-up, limited number of progression events, and potential residual confounding, these findings should be interpreted as hypothesis-generating. Future prospective multicenter studies are required to determine whether psychological assessment provides incremental prognostic value beyond conventional clinical and radiographic factors and whether targeted perioperative interventions improve clinical outcomes or structural survivorship after mUKA.

## Figures and Tables

**Table 1:** Inclusion and exclusion criteria of the study.

Inclusion Criteria	Exclusion Criteria
(1) Diagnosed with anteromedial osteoarthritis (AMOA) or spontaneous osteonecrosis of the knee (SONK)	(1) Incomplete imaging data (lack of preoperative X-rays, MRI, or at least one postoperative full-length lower-limb X-ray) or incomplete clinical outcomes
(2) Met UKA indications: severe knee joint pain; AMOA with “bone-on-bone” wear or SONK; intact anterior cruciate ligament (ACL) functionality; flexion contracture < 15°; absence of patellofemoral joint severe wear, subluxation or lateral groove deformity; and intraoperative lateral condyle cartilage Outerbridge Grade ≤ II	(2) History of joint replacement or fracture on the operated limb
	(3) Preoperative MRI indicating lateral meniscus extrusion
	(4) Identified as having suboptimal UKA due to overcorrection

**Table 2:** Comparison of baseline characteristics between the AD and Non-AD groups.

Variables	AD (*n* = 26)	Non-AD (*n* = 177)	*p*
Diagnosis			0.425
OA	25 (96.2%)	174 (98.3%)	
SONK	1 (3.8%)	3 (1.7%)	
Age, years	71.12 ± 7.37	67.9 ± 6.96	**0.030**
BMI, kg/m^2^	27.53 ± 3.58	27.51 ± 3.57	0.983
Gender			0.239
Female	23 (88.5%)	139 (78.5%)	
Male	3 (11.5%)	38 (21.5%)	
Side			0.549
L	11 (42.3%)	86 (48.6%)	
R	15 (57.7%)	91 (51.4%)	
Preoperative HKA, °	171.23 ± 4.37	171.89 ± 3.73	0.412
Postoperative HKA, °	175.66 ± 2.36	176.04 ± 2.92	0.523
Follow-up, months	74.27 ± 14.17	70.25 ± 14.72	0.194

Data are expressed as the mean value and standard deviation for continuous variables and numbers for categorical variables. AD indicates anxiety and/or depression; OA, osteoarthritis; SONK, Spontaneous osteonecrosis of the knee; BMI, body mass index; HKA indicates hip–knee–ankle angle. Bold *p* values indicate statistical significance.

**Table 3:** Comparison of clinical outcomes and complications at 5 years after UKA between the AD and Non-AD groups.

Variables	AD (*n* = 26)	Non-AD (*n* = 177)	*p*
Preoperative OKS	26.12 ± 5.88	24.99 ± 6.08	0.377
Postoperative OKS	33.81 ± 6.31	42.31 ± 3.67	**<0.001**
Change in OKS	7.69 ± 6.92	17.32 ± 6.40	**<0.001**
Postoperative FJS	60.69 ± 27.60	87.94 ± 15.12	**<0.001**
Postoperative Kujala	49.69 ± 14.17	65.85 ± 11.20	**<0.001**
Perioperative complications	5 (19.2%)	17 (9.6%)	0.171
Wound healing	1 (3.8%)	4 (2.6%)	
Periprosthetic joint infection	1 (3.8%)	2 (1.1%)	
Urinary tract infection	1 (3.8%)	5 (2.8%)	
Deep vein thrombosis	1 (3.8%)	4 (2.3%)	
Delirium	1 (3.8%)	2 (1.1%)	

Data are expressed as the mean value and standard deviation for continuous variables and numbers for categorical variables. AD indicates anxiety and/or depression; OKS, Oxford Knee Score; FJS, Forgotten Joint Score. Bold *p* values indicate statistical significance.

**Table 4:** Comparison of lateral compartment osteoarthritis progression at 5 years after UKA between the AD and Non-AD groups.

	AD (*n* = 15)	Non-AD (*n* = 84)	*p*
K/L grade within one week postoperatively			0.687
0	4 (26.7%)	32 (38.1%)	
I	9 (60%)	43 (51.2%)	
II	2 (13.3%)	9 (10.7%)	
K/L grade at final follow-up			**0.005**
0	0 (0%)	16 (19.0%)	
I	9 (60%)	59 (70.3%)	
II	5 (33.3%)	9 (10.7%)	
III	1 (6.7%)	0 (0%)	
Lateral compartment osteoarthritis progression			
Yes	9 (60%)	17 (20.2%)	**0.004**
No	6 (40%)	67 (79.8%)	

Data are expressed as numbers for categorical variables. AD indicates anxiety and/or depression; K/L, Kellgren–Lawrence. Bold *p* values indicate statistical significance.

**Table 5:** Characteristics associated with radiographic lateral compartment osteoarthritis progression.

Risk Factors	Univariate Analysis			Multivariate Analysis *		
	LOP (*n* = 26)	Not-LOP (*n* = 73)	*p*	aOR	95% CI	*p*
**Baseline characteristics**						
AD	9 (34.6%)	6 (8.2%)	**0.004**	7.453	2.033–27.082	**0.003**
Age, years	67.69 ± 7.25	67.53 ± 6.32	0.916	1.012	0.928–1.089	0.745
BMI, kg/m^2^	27.51 ± 3.47	26.89 ± 2.66	0.348			
Gender						
Female	20 (76.9%)	57 (78.1%)	0.903			
Male	6 (23.1%)	16 (21.9%)				
Side			**0.008**	4.180	1.370–12.751	**0.012**
L	6 (23.1%)	39 (53.4%)				
R	20 (76.9%)	34 (46.6%)				
Preoperative HKA, °	172.65 ± 4.04	171.79 ± 4.02	0.351			
Preoperative OKS	25.69 ± 6.42	25.1 ± 5.16	0.637			
**Perioperative and early postoperative characteristics**						
Postoperative HKA, °	177.13 ± 2.54	175.67 ± 3.09	**0.033**	1.240	1.025–1.516	**0.031**
Perioperative complications	3 (11.5%)	7 (9.6%)	1.000			
Follow-up radiographic and clinical outcomes						
HKA at the 5-year follow-up, °	175.70 ± 3.24	174.21 ± 3.06	**0.037**			
Change in HKA at follow-up, °	1.43 ± 1.69	1.47 ± 1.65	0.924			
Follow-up, months	69.5 ± 13.09	76.23 ± 14.64	0.051			
Postoperative OKS	40.85 ± 5.21	43.38 ± 3.47	**0.006**			
Change in OKS	15.15 ± 7.14	18.29 ± 5.48	**0.023**			
Postoperative FJS	83.42 ± 17.93	87.75 ± 15.73	0.248			
Postoperative Kujala	62.73 ± 10.78	67.32 ± 10.62	**0.048**			

Data are expressed as the mean value and standard deviation for continuous variables and numbers for categorical variables. * Covariates were selected a priori according to clinical relevance, previous evidence, and univariable findings, while limiting the number of parameters because of the small number of outcome events. AD indicates anxiety and/or depression; OA, osteoarthritis; SONK, Spontaneous osteonecrosis of the knee; BMI, body mass index; HKA indicates hip–knee–ankle angle; OKS, Oxford Knee Score; FJS, Forgotten Joint Score. Bold *p* values indicate statistical significance.

## Data Availability

The data can be obtained through the email under reasonable request: huangyekps@163.com.

## Supplementary Materials

The following supporting information can be downloaded at: https://www.mdpi.com/article/10.3390/jcm15176568/s1, Table S1. Comparison of baseline characteristics between the CRF and IRF groups. Table S2. Primary and sensitivity multivariable logistic regression models for LOP after mUKA. Table S3. Follow-up-adjusted sensitivity analysis of factors associated with lateral compartment osteoarthritis progression after mUKA. Table S4. Model diagnostics for the primary multivariable logistic regression model. Table S5. Interobserver reliability of radiographic assessment. STROBE Statement—Checklist of items that should be included in reports of cohort studies.
