# Supplementary material for: Preoperative Anxiety and Depression Are Associated with Lateral Compartment Osteoarthritis Progression After Medial Unicompartmental Knee Arthroplasty: A Retrospective Observational Cohort Study
Source: J Clin Med. 2026 Aug 26;15(17):6568. doi: 10.3390/jcm15176568 (PMC13566752; doi:10.3390/jcm15176568)
Supplement: Supplementary file 1 [file jcm-15-06568-s001.zip › jcm-4426393-supplementary.pdf]

Table S1. Comparison of baseline characteristics between the CRF and IRF groups

Variables	CRF (n=99)	IRF (n=104)	Standardized difference	p
Diagnosis			0.06	0.812
OA	97 (98.0%)	102 (98.1%)		
SONK	2 (2.0%)	2 (1.9%)		
Age, years	67.60 ± 6.67	68.80 ± 7.46	−0.17	0.271
BMI, kg/m²	27.15 ± 2.96	27.82 ± 3.92	−0.19	0.142
Female sex	77 (77.8%)	85 (81.7%)	−0.10	0.489
Right-sided surgery	55 (55.6%)	51 (49.0%)	0.13	0.356
AD	15 (15.2%)	11 (10.6%)	0.14	0.330
Preoperative HKA, °	172.04 ± 4.03	171.58 ± 3.74	0.12	0.445
Postoperative HKA, °	176.12 ± 3.01	175.87 ± 2.82	0.09	0.681
Preoperative OKS	25.31 ± 6.04	24.91 ± 6.02	0.07	0.644
Postoperative OKS	40.75 ± 5.32	41.61 ± 4.47	−0.18	0.214
Change in OKS	15.44 ± 7.13	16.70 ± 6.65	−0.18	0.301
Postoperative FJS	83.12 ± 20.47	85.91 ± 17.36	−0.15	0.267
Postoperative Kujala score	62.84 ± 13.18	64.56 ± 11.97	−0.14	0.334
Overall complications	10 (10.1%)	12 (11.5%)	−0.05	0.747
Follow-up, months	74.43 ± 14.46	67.44 ± 14.14	0.49	0.001

Data are expressed as mean ± standard deviation or number (percentage), as appropriate. Standardized differences were calculated to describe the magnitude of between-group differences; CRF, complete radiographic follow-up; IRF, incomplete radiographic follow-up; OA, osteoarthritis; SONK, spontaneous osteonecrosis of the knee; BMI, body mass index; AD, anxiety and/or depression; HKA, hip–knee–ankle angle; OKS, Oxford Knee Score; FJS, Forgotten Joint Score. Bold p values indicate statistical significance.

Table S2. Primary and sensitivity multivariable logistic regression models for LOP after mUKA

**Variables**	**Primary model[1]**		**Expanded sensitivity model[2]**		**Parsimonious sensitivity model[3]**	
	**aOR (95% CI)**	**p**	**aOR (95% CI)**	**p**	**aOR (95% CI)**	**p**
Anxiety and/or depression	7.453 (2.033–27.082)	0.003	6.184 (1.742–21.952)	0.005	5.936 (1.823–19.341)	0.004
Age (years)	1.012 (0.928–1.089)	0.745	1.019 (0.952–1.091)	0.584	—	—
Female sex	—	—	1.271 (0.382–4.229)	0.695	—	—
BMI (kg/m^2)	—	—	1.061 (0.922–1.221)	0.409	—	—
Right-sided surgery	4.180 (1.370–12.751)	0.012	3.604 (1.172–11.085)	0.026	3.781 (1.253–11.409)	0.019
Immediate postoperative HKA (°)	1.240 (1.025–1.516)	0.031	1.218 (1.012–1.467)	0.037	1.229 (1.031–1.466)	0.022

aOR, adjusted odds ratio; CI, confidence interval; BMI, body mass index; HKA, hip–knee–ankle angle.

[1]Primary model: prespecified model including anxiety and/or depression, age, surgical side, and immediate postoperative HKA.

[2]Expanded model: primary model with additional adjustment for sex and BMI.

[3]Parsimonious model: model including the prespecified core variables (anxiety and/or depression, surgical side, and immediate postoperative HKA) to evaluate the robustness of the primary findings while minimizing model complexity.

Table S3. Follow-up-adjusted sensitivity analysis of factors associated with lateral compartment osteoarthritis progression after mUKA

**Variables**	**aOR (95% CI)**	**p**
AD	6.312 (1.707–23.423)	**0.006**
Age	1.023 (0.962–1.090)	0.487
Right-sided surgery	3.744 (1.222–11.475)	**0.021**
Postoperative HKA	1.213 (1.015–1.456)	**0.041**
Follow-up duration, per 10 months	1.294 (0.941–1.777)	0.116

aOR, adjusted odds ratio; CI, confidence interval; AD, anxiety and/or depression; HKA, hip − knee − ankle angle. Bold p values indicate statistical significance.

Table S4. Model diagnostics for the primary multivariable logistic regression model

**Parameter**	**Value**
Hosmer–Lemeshow goodness-of-fit test	p=0.713
Nagelkerke R^2	0.315
AUROC (95% CI)	0.797 (0.699–0.895)
Variance inflation factor (VIF) range	1.08–1.41
Maximum VIF	1.41
Maximum Cook's distance	0.173
Criterion for influential observations	Cook's distance >1.0
Influential observations identified	None

AUROC, area under the receiver operating characteristic curve; VIF, variance inflation factor.

Table S5. Interobserver reliability of radiographic assessment

Parameter	Value
HKA measurements ICC (95% CI)	
Preoperative HKA	0.91 (0.87–0.94)
Postoperative HKA	0.89 (0.84–0.93)
Kellgren–Lawrence grading	
Weighted κ (95% CI)	0.84 (0.75–0.92)
Initial discrepant readings	11/99 (11.1%)
Cases requiring senior adjudication	3/99 (3.0%)

ICC, intraclass correlation coefficient; HKA, hip–knee–ankle angle. Two experienced musculoskeletal radiologists independently evaluated all radiographs. Disagreements in HKA measurements were averaged after joint review, whereas disagreements in Kellgren–Lawrence grading were resolved by consensus. Cases without consensus were adjudicated by a senior musculoskeletal radiologist.

STROBE Statement—Checklist of items that should be included in reports of ***cohort studies***

	Item No	Recommendation	Page No
**Title and abstract**	1	(*a*) Indicate the study's design with a commonly used term in the title or the abstract	2
		(*b*) Provide in the abstract an informative and balanced summary of what was done and what was found	2
**Introduction**			
Background/rationale	2	Explain the scientific background and rationale for the investigation being reported	2
Objectives	3	State specific objectives, including any prespecified hypotheses	3
**Methods**			
Study design	4	Present key elements of study design early in the paper	3
Setting	5	Describe the setting, locations, and relevant dates, including periods of recruitment, exposure, follow-up, and data collection	3
Participants	6	(*a*) Give the eligibility criteria, and the sources and methods of selection of participants. Describe methods of follow-up	3
		(*b*) For matched studies, give matching criteria and number of exposed and unexposed	3
Variables	7	Clearly define all outcomes, exposures, predictors, potential confounders, and effect modifiers. Give diagnostic criteria, if applicable	3
Data sources/ measurement	8*	For each variable of interest, give sources of data and details of methods of assessment (measurement). Describe comparability of assessment methods if there is more than one group	4
Bias	9	Describe any efforts to address potential sources of bias	4
Study size	10	Explain how the study size was arrived at	4
Quantitative variables	11	Explain how quantitative variables were handled in the analyses. If applicable, describe which groupings were chosen and why	5
Statistical methods	12	(*a*) Describe all statistical methods, including those used to control for confounding	5
		(*b*) Describe any methods used to examine subgroups and interactions	5
		(*c*) Explain how missing data were addressed	5
		(*d*) If applicable, explain how loss to follow-up was addressed	5
		(*e*) Describe any sensitivity analyses	5
**Results**			
Participants	13*	(a) Report numbers of individuals at each stage of study—eg numbers potentially eligible, examined for eligibility, confirmed eligible, included in the study, completing follow-up, and analysed	6
		(b) Give reasons for non-participation at each stage	6
		(c) Consider use of a flow diagram	6
Descriptive data	14*	(a) Give characteristics of study participants (eg demographic, clinical, social) and information on exposures and potential confounders	6
		(b) Indicate number of participants with missing data for each variable of interest	6
		(c) Summarise follow-up time (eg, average and total amount)	6
Outcome data	15*	Report numbers of outcome events or summary measures over time	7

Main results	16	(*a*) Give unadjusted estimates and, if applicable, confounder-adjusted estimates and their precision (eg, 95% confidence interval). Make clear which confounders were adjusted for and why they were included	6-7
		(*b*) Report category boundaries when continuous variables were categorized	7
		(*c*) If relevant, consider translating estimates of relative risk into absolute risk for a meaningful time period	7
Other analyses	17	Report other analyses done—eg analyses of subgroups and interactions, and sensitivity analyses	7
**Discussion**			
Key results	18	Summarise key results with reference to study objectives	7
Limitations	19	Discuss limitations of the study, taking into account sources of potential bias or imprecision. Discuss both direction and magnitude of any potential bias	9
Interpretation	20	Give a cautious overall interpretation of results considering objectives, limitations, multiplicity of analyses, results from similar studies, and other relevant evidence	8
Generalisability	21	Discuss the generalisability (external validity) of the study results	7-9
**Other information**			
Funding	22	Give the source of funding and the role of the funders for the present study and, if applicable, for the original study on which the present article is based	10

*Give information separately for exposed and unexposed groups.

Note: An Explanation and Elaboration article discusses each checklist item and gives methodological background and published examples of transparent reporting. The STROBE checklist is best used in conjunction with this article (freely available on the Web sites of PLoS Medicine at http://www.plosmedicine.org/, Annals of Internal Medicine at http://www.annals.org/, and Epidemiology at http://www.epidem.com/). Information on the STROBE Initiative is available at http://www.strobe-statement.org.
